# Have Middle-Aged and Older Americans Become Lonelier? 20-Year Trends From the Health and Retirement Study

**DOI:** 10.1093/geronb/gbad062

**Published:** 2023-06-04

**Authors:** Daniel L Surkalim, Philip J Clare, Robert Eres, Klaus Gebel, Adrian Bauman, Ding Ding

**Affiliations:** Sydney School of Public Health, Faculty of Medicine and Health, The University of Sydney, Camperdown, New South Wales, Australia; Charles Perkins Centre (D17), The University of Sydney, Camperdown, New South Wales, Australia; Sydney School of Public Health, Faculty of Medicine and Health, The University of Sydney, Camperdown, New South Wales, Australia; National Drug and Alcohol Research Centre, UNSW Sydney, Sydney, New South Wales, Australia; Neurodisability and Rehabilitation, Murdoch Children’s Research Institute, Parkville, Victoria, Australia; Department of Paediatrics, The University of Melbourne, Parkville, Victoria, Australia; School of Public Health, Faculty of Health, University of Technology Sydney, Ultimo, New South Wales, Australia; Sydney School of Public Health, Faculty of Medicine and Health, The University of Sydney, Camperdown, New South Wales, Australia; Charles Perkins Centre (D17), The University of Sydney, Camperdown, New South Wales, Australia; Sydney School of Public Health, Faculty of Medicine and Health, The University of Sydney, Camperdown, New South Wales, Australia; Charles Perkins Centre (D17), The University of Sydney, Camperdown, New South Wales, Australia

**Keywords:** Loneliness, Prevalence, Social epidemiology, Social health, Social well-being

## Abstract

**Objectives:**

Despite media and public dialog portraying loneliness as a worsening problem, little is known about how the prevalence of loneliness has changed over time. Our study aims to identify (a) temporal trends in episodic and sustained loneliness (lonely in 1 wave vs consistently lonely in 3 consecutive waves); (b) trends across sociodemographic subgroups by sex, race/ethnicity, birth cohort, education, employment status, marital status, and living alone; and (c) longitudinal predictors of loneliness in middle-aged and older Americans (≥50 years).

**Methods:**

Based on Waves 3 (1996) to 14 (2018) of the Health and Retirement Study (*n* = 18,841–23,227), we conducted a series of lagged mixed-effects Poisson regression models to assess trends of episodic and sustained loneliness in the overall and sociodemographic subgroup samples (by sex, race/ethnicity, birth cohort, education, employment, relationship, and living alone status). To examine the predictors of episodic and sustained loneliness, we used a multivariate mixed-effects Poisson regression model with all sociodemographic variables entered into the same model.

**Results:**

Episodic loneliness prevalence decreased from 20.1% to 15.5% and sustained loneliness from 4.6% to 3.6%. Trends were similar across most subgroups. Males, Caucasians, those born in 1928–1945, with university education, working, married/partnered, and those not living alone reported lower episodic and sustained loneliness, although associations with sustained loneliness were stronger.

**Discussion:**

Contrary to common perceptions, loneliness has decreased over 20 years of follow-up in middle-aged and older Americans. Several sociodemographic subgroups have been identified as having a higher risk of loneliness, prompting targeted public health attention.

Loneliness is defined as a negative subjective feeling resulting from the discrepancy between a person’s desired and actual social relationships (Peplau & Perlman, 1982). Transient loneliness is a common and natural human experience, often considered as a drive to connect. When loneliness is experienced for a prolonged period, it could lead to physical, social, and mental health problems (Lim et al., 2020), including poorer subjective well-being, substance abuse, and depressive symptomatology (Leigh-Hunt et al., 2017). A recent systematic review revealed that problematic levels of loneliness, defined by chronicity and severity, are prevalent around the world, and particularly common among older adults (Surkalim et al., 2022).

Considered to be an epidemic by many health professionals, including the U.S. Surgeon General, Vivek Murthy (Murthy, 2017), population awareness of loneliness has increased over time. During the coronavirus disease 2019 (COVID-19) pandemic, loneliness has become a commonly discussed topic as isolation and physical distancing have disrupted social connections (Clair et al., 2021; Smith & Lim, 2020; Stefana et al., 2020). Although media and public dialogue tend to portray loneliness as a worsening social problem (Beaton, 2017; Coombs, 2020), few empirical studies have examined such claims. Despite a number of global prevalence studies available, inconsistent measurement tools, study periods, and population samples have made it difficult to identify trends for specific populations over time (Surkalim et al., 2022). For example, two studies that examined temporal trends found little evidence that loneliness levels have increased over time in American (Hawkley et al., 2019) and Swedish (Dahlberg et al., 2018) samples of older adults. There appears to be a mismatch between the public perception of and the empirical evidence for the temporal trends in loneliness.

Much of the research works on loneliness have used measures reporting loneliness at one point in time, which cannot distinguish those who experience transient feelings of loneliness from those who are chronically lonely. The former is a common human experience, whereas the latter is a health and social concern. According to the conceptual framework by Lim et al. (2020), the experience of loneliness results from risk factors (e.g., demographic characteristics, chronic health conditions, social environments) interacting with triggering events (e.g., retirement and marital disruption). Therefore, one may expect that demographic and social trends (e.g., population aging and women’s increasing participation in the workforce) and changes in social environments (e.g., mobile technology and social media) could lead to changes in both risk factors and triggers and consequentially affect the prevalence of loneliness in both the general population and specific subpopulations. Several risk factors have been identified in previous systematic reviews (Cohen-Mansfield et al., 2016; Dahlberg et al., 2022): namely, a higher risk of loneliness among individuals who are older, female, nonmarried/partnered, not working, living alone, with lower income, self-rated social network quality, self-perceived health, or functional status.

In this study, we aimed to address several of the evidence gaps described earlier. Based on Lim and colleagues’ conceptual model of loneliness (Lim et al., 2020), using data from the Health and Retirement Study (HRS; Sonnega et al., 2014), we (a) identified and compared the temporal trends in loneliness over 20 years based on measures at one point in time (episodic prevalence) and over a sustained period (sustained prevalence), (b) examined temporal trends in loneliness across different population subgroups, and (c) identified sociodemographic predictors of loneliness for middle-aged and older Americans (≥50 years).

## Method

### Sampling and Procedure

Data are from Waves 3 (1996) to 14 (2018) of the HRS, where loneliness data from Waves 4 to 14 and sociodemographic variables from Waves 3 to 13 were used (*n* = 18,841–23,227). The HRS is a nationally representative, longitudinal study of noninstitutionalized American adults born in 1965 or earlier. Surveys were administered biennially, gathering a wide range of information such as respondents’ demographic characteristics, social relationships, employment, income, and wealth. Each cohort sample was created using a stratified, multistage probability design, oversampling African Americans and Hispanics. Additional birth cohorts were added to the sample in 1998, 2004, 2010, and 2016 (Supplementary Figure 1; Sonnega et al., 2014). Sampling weights were updated for each wave to account for attrition. Details of the HRS have been provided elsewhere (Weir, 2017). We did not include loneliness measures from the first three waves because loneliness was measured using a different question in Wave 1 (1992) and the samples for Waves 1 and 2 are not comparable with the later samples, due to the introduction of younger cohort groups later on (Health and Retirement Study, 2017). Finally, we did not include Wave 15 (2020) because the loneliness measure used was not comparable with the earlier waves.

Briefly, the study was conducted in compliance with the relevant local Institutional Review Board (Sonnega et al., 2014). Participants were provided with an informed consent information document and were instructed to read a confidentiality statement before each interview (Weir, 2017). Participation in the study required verbal consent, as well as written authorization for social security administration linkage, biomarker and physical measure collection, and proxy respondents for vulnerable populations (Weir, 2017). Between 1996 and 2004, interviews were predominantly conducted via telephone, except for older participants (≥80 years) who were administered face-to-face interviews at each wave (Fisher & Ryan, 2017). From 2006 onwards, half of the sample was interviewed via telephone whereas the other half received a face-to-face interview, with each half alternating interview mode at every wave (Fisher & Ryan, 2017).

This study follows the Strengthening the Reporting of Observational studies in Epidemiology guidelines for cohort studies (Von Elm et al., 2007). Full details can be found in Supplementary Table 1.

### Measures

#### Loneliness

The outcome of loneliness was measured using a single-item measure of loneliness adapted from an item within the Center for Epidemiological Studies—Depression Scale (Radloff, 1977): “(Much of the time during the past week…) You felt lonely: (1) Yes; (2) No.”

The prevalence of loneliness is operationalized as “episodic” and “sustained” prevalence. “Episodic loneliness” captures the transient experience of loneliness and is defined as reporting feeling lonely within a 1-week recall period at the time of survey completion. “Sustained loneliness” captures the chronic experience of loneliness and is defined as reporting feeling lonely in three consecutive survey waves. Three waves were chosen to define the “sustained” period as three are the minimal data points required to ascertain trends.

#### Sociodemographic characteristics

Based on Lim and colleagues’ conceptual model of loneliness (Lim et al., 2020) and supporting literature (Cohen-Mansfield et al., 2016; Dahlberg et al., 2022), the following sociodemographic characteristics were considered important, either as potential predictors of loneliness or effect modifiers of the temporal trends: sex (male, female), race/ethnicity (Hispanic, Caucasian, African American, Other), birth cohort (the Greatest Generation [born 1901–1927], the Silent Generation [born 1928–1945], Baby Boomers [born 1946–1964]), education (less than high school, high school/General Educational Development, some college, college or higher), employment status (working, retired, unemployed), marital status (married/partnered, single/divorced/separated, widowed), and living alone status (yes/no).

### Analysis

To show the composition of the sample, we calculated unweighted, descriptive statistics of the sample using numbers and percentages across a range of sociodemographic variables. We then estimated the raw population prevalence of both episodic and sustained loneliness for each wave between Wave 4 (1998) and Wave 14 (2018), using a weighted percentage.

To model the trends over time, we conducted a series of mixed-effects Poisson regression models to account for the correlation across repeated measures within participants over time (Zou, 2004). We tested the main effects of the survey wave (as a continuous variable) on episodic and sustained loneliness as the dependent variables. We assessed a simple linear term against both quadratic and cubic functions, comparing model fit using Akaike information criterion and Bayes information criterion (BIC). For both episodic and sustained loneliness, the linear trend showed the best model fit based on BIC. For episodic loneliness, the quadratic term marginally improved model fit, but only by 0.004%, which was not sufficient to justify the extra complexity of the model.

To determine whether the trend in loneliness differs by sociodemographic subgroups over time, in the model we included survey wave and one sociodemographic characteristic at a time (including sex, race/ethnicity, birth cohort, education, employment status, marital status, living alone status) as the independent variables, and loneliness as the dependent variable, with an addition of a multiplicative interaction term between wave and each of the sociodemographic characteristics. From these models, we assessed trends in each of the sociodemographic subgroups and presented them in graphs. Finally, to examine the predictors of episodic and sustained loneliness over time, we used a multivariate mixed-effects Poisson regression model for both loneliness outcomes, with all sociodemographic variables entered into the same model as independent variables (Zou, 2004).

For all analyses, we used lagged models with sociodemographic variables from one wave predicting loneliness in the subsequent wave(s). For example, for episodic prevalence of loneliness, data from Wave 4 were used to predict loneliness outcomes in Wave 5, and Wave 5 predicted Wave 6. For sustained loneliness, data from one wave were used to predict loneliness in the subsequent period of three waves. For example, sociodemographic characteristics at Wave 4 were used to predict “sustained loneliness” patterns derived from data at Waves 5–7. When calculating sustained loneliness using unimputed data, participants were only coded if they had data for three consecutive waves. For example, if a participant had data for Waves 5 and 6 but not Wave 7, their sustained loneliness for that period would be coded as missing. Further details of the models are included in Supplementary Appendix A.

Prevalence is reported as weighted percentages. The results of the mixed-effects Poisson regression model are presented as marginal predicted prevalence with their corresponding 95% confidence intervals (95% CIs). Where rounding of the bounds of the CI makes it unclear whether the interval crosses the null, we also report the *p* value. All analyses were conducted in Stata 17.0 (StataCorp, 2019) and R 4.2.1 (R Core Team, 2020).

#### Missing data

There were between 14.3% and 38.7% missing data in Waves 3–14 (patterns of missing data in Supplementary Appendix B). As such, to reduce the possibility of bias where missingness is not completely at random (Pedersen et al., 2017), we conducted analyses using multiple imputation (White et al., 2011), with imputation conducted using fully conditional specification (Huque et al., 2018), using random forests (Shah et al., 2014). To be conservative, we used *M* = 50 imputations (White et al., 2011). More details on the imputation are included in Supplementary Appendix C. Sample characteristics and raw trends were examined using the unimputed data, whereas modeled trends and the multivariable models were conducted on each imputed data set and combined using Rubin’s rules (Rubin, 2004).

## Results

### Descriptive Statistics

At Wave 3 (1996; *n* = 19,253), participants were 57.6% female with the majority born in the United States (89.6%). Participants were predominantly Caucasian (75.9%), married (69.7%), and of the Silent Generation (born 1928–1945; 57.1%). Nearly half (47.0%) were retired, with 39.6% still working, and 20.7% living alone. Detailed unweighted sociodemographic characteristics for each wave are provided in Table 1.

**Table 1.** Sample Characteristics of the Health and Retirement Study (1996–2018)

	Year	1996	1998	2000	2002	2004	2006	2008	2010	2012	2014	2016	2018
	Sample size^a^	*n =* 19,253	*n* = 22,640	*n* = 21,253	*n* = 19,733	*n* = 21,817	*n* = 20,064	*n* = 18,841	*n* = 23,227	*n* = 21,836	*n* = 20,405	*n* = 21,775	*n* = 20,853
Sex	Male	42.4	42.5	41.9	41.3	41.9	41.5	41.4	42.5	42.2	41.9	42.7	42.5
	Female	57.6	57.5	58.1	58.7	58.1	58.5	58.6	57.5	57.8	58.1	57.3	57.5
Race/	White/Caucasian	75.9	76.3	76.2	76.2	74.1	74.2	73.9	65.8	65.2	64.1	60.5	59.5
ethnicity	Black/African American	14.3	13.6	13.5	13.4	13.9	13.9	13.9	18.0	18.2	18.7	20.0	20.3
	Hispanic	7.9	8.0	8.1	8.3	9.5	9.4	9.6	12.9	13.2	13.7	15.1	15.6
	Other	1.8	2.0	2.1	2.1	2.5	2.5	2.6	3.3	3.3	3.4	4.5	4.6
Birth cohort	The Greatest Generation (1901–1927)	39.0	34.5	31.8	28.9	22.2	19.5	16.8	10.3	8.5	6.7	4.1	3.0
	The Silent Generation (1928–1945)	57.1	56.6	58.5	60.6	53.4	54.3	55.3	42.4	41.9	41.0	34.3	32.9
	Baby Boomers (1946–1964)	3.8	8.9	9.6	10.5	24.3	26.3	27.9	47.2	49.6	52.4	61.6	64.1
Education	Less than high school	31.2	28.1	27.1	26.0	23.2	22.2	21.3	19.4	18.7	18.4	17.1	16.7
	High school graduate/GED	35.6	35.6	35.8	36.2	35.3	35.3	35.4	34.2	34.1	33.6	32.9	32.7
	Some college	18.1	19.2	19.4	19.7	21.5	21.7	22.0	24.3	24.6	25.0	26.1	26.4
	College and above	15.2	17.2	17.7	18.2	20.0	20.8	21.3	22.1	22.6	23.0	23.9	24.2
Employment	Working	39.6	41.0	38.3	34.6	40.0	36.9	34.8	40.9	39.3	37.0	41.0	37.3
status	Retired	47.0	46.0	47.1	50.4	48.5	53.0	56.0	49.4	53.3	57.2	52.7	57.1
	Unemployed	13.4	13.0	14.6	15.1	11.6	10.1	9.2	9.8	7.4	5.8	6.3	5.6
Marital	Married/partnered	69.7	68.8	67.5	65.9	66.8	65.6	64.3	64.4	63.5	61.8	60.4	59.8
status	Single/divorced/separated	10.7	11.8	11.7	11.9	13.5	13.5	14.0	18.2	18.3	18.6	21.8	22.1
	Widowed	19.6	19.4	20.8	22.1	19.7	20.9	21.7	17.4	18.2	19.5	17.8	18.1
Live alone	Yes	20.7	21.0	22.4	23.7	22.2	24.3	24.9	23.0	23.7	24.7	24.7	25.9
	No	79.4	79.0	77.6	76.3	77.8	75.7	75.1	77.0	76.3	75.3	75.4	74.1
Born in US	Yes	89.6	90.0	89.9	89.8	89.0	89.0	88.9	85.8	85.6	85.1	84.1	83.7
	No	10.4	10.0	10.1	10.2	11.0	11.0	11.1	14.2	14.4	14.9	15.9	16.3

*Notes*: Percentages may not add up to 100.0% due to rounding. GED = General Educational Development.

^a^Specific sample sizes may differ for each demographic variable as a result of missing data.

### Prevalence of Loneliness over Time

**Figure 1.**
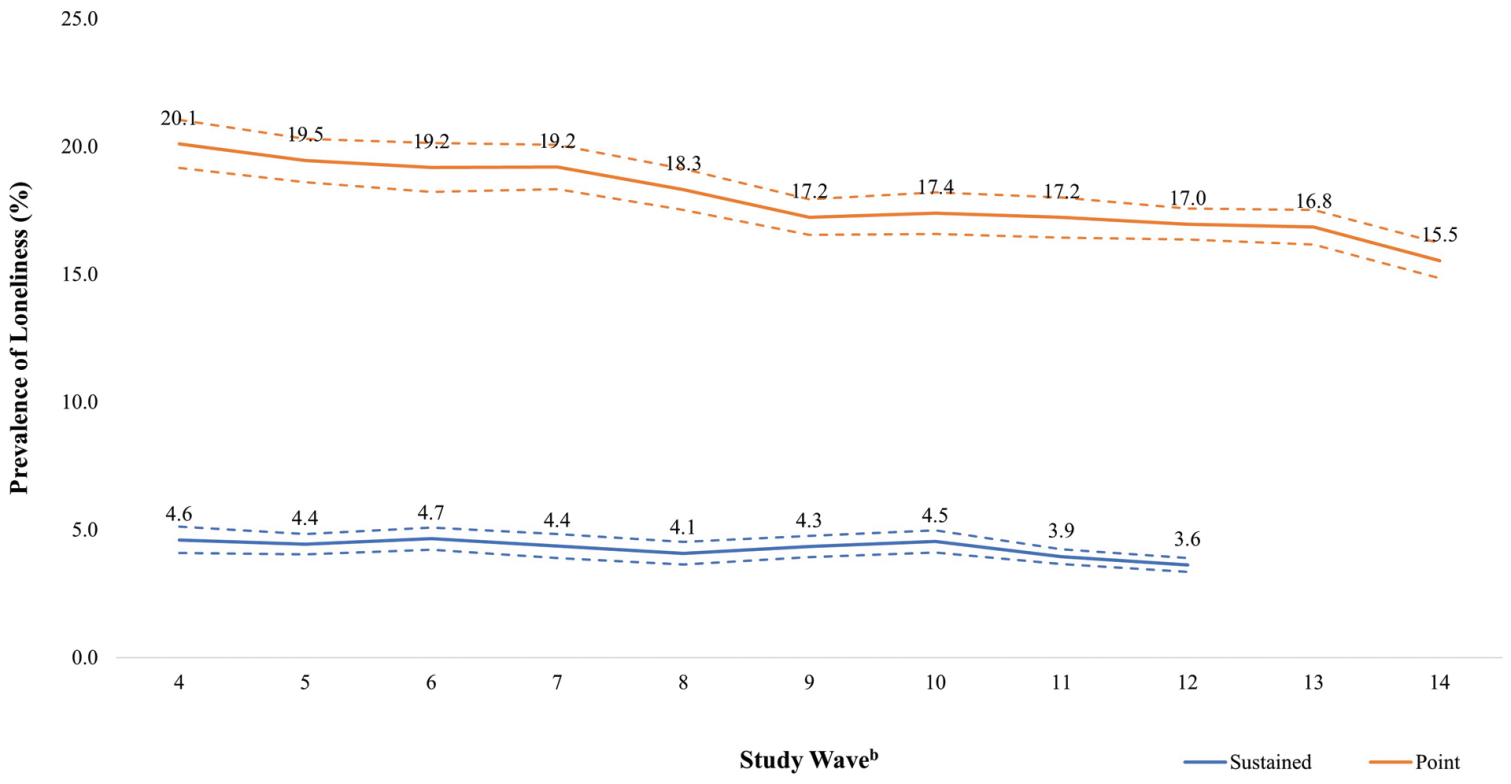
Prevalence trends for episodic and sustained loneliness^a^ with confidence intervals. Prevalence calculated as the weighted mean in each wave. ^a^Data available for episodic (*n* = 232,436–232,513) and sustained (*n* = 162,820–162,920) loneliness. ^b^For sustained loneliness, “study wave” indicates three consecutive Health and Retirement Study waves from which a sustained loneliness measure could be derived (e.g., Wave 4 refers to Waves 4–6, Wave 12 refers to Waves 12–14).

Figure 1 displays overall trends in loneliness across all wave years. The prevalence estimates provided account for the clustering of measures within individual participants. The prevalence of episodic loneliness decreased from 20.1% in 1998 to 15.5% in 2018. The prevalence of sustained loneliness decreased from 4.6% in the 1998–2002 period to 3.6% in 2014–2018.

### Temporal Trends in Loneliness

**Figure 2.**
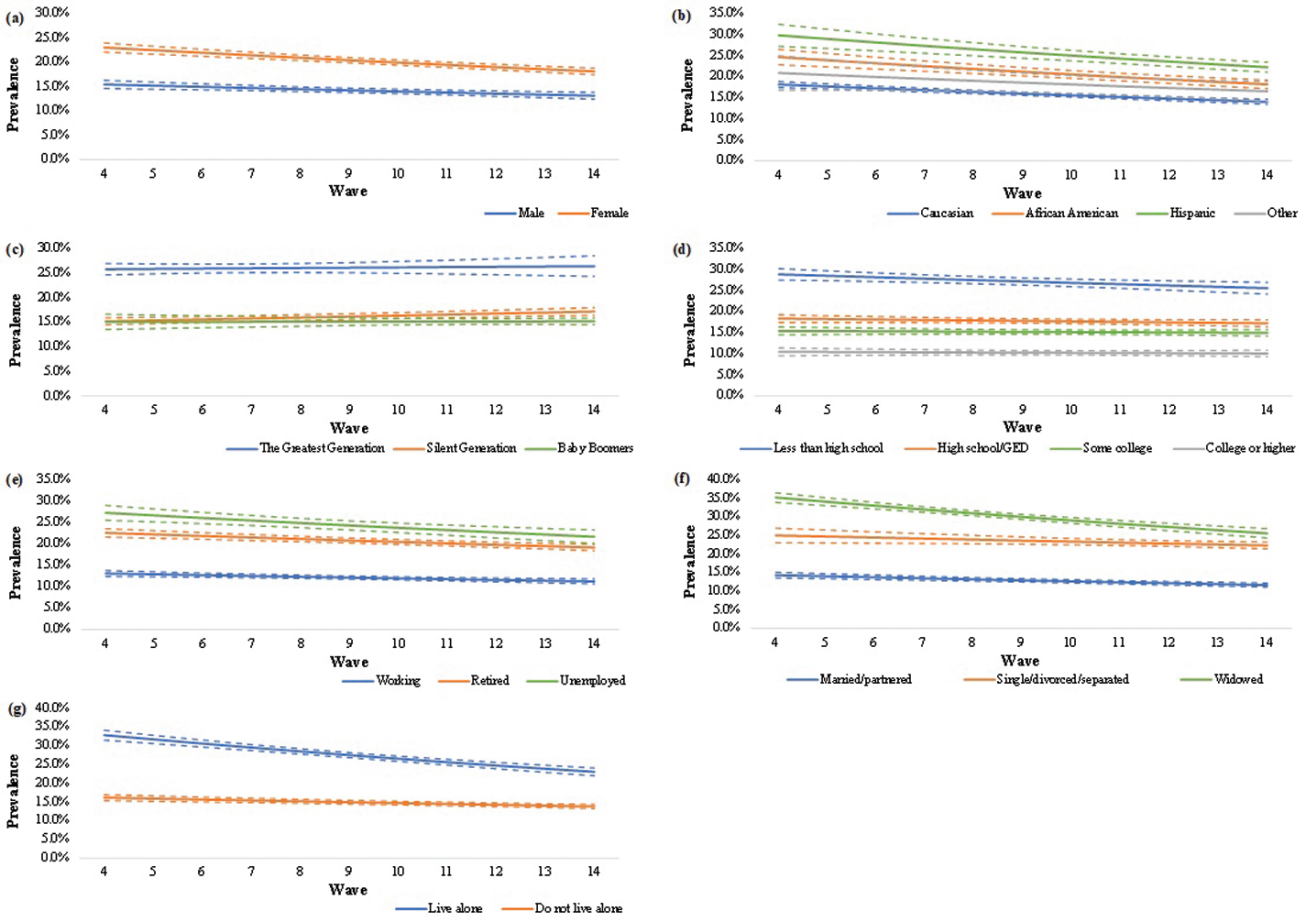
Prevalence of episodic loneliness across (A) sex, (B) race/ethnicity, (C) birth cohort, (D) education, (E) employment, (F) marital status, and (G) living alone with 95% confidence intervals.

**Figure 3.**
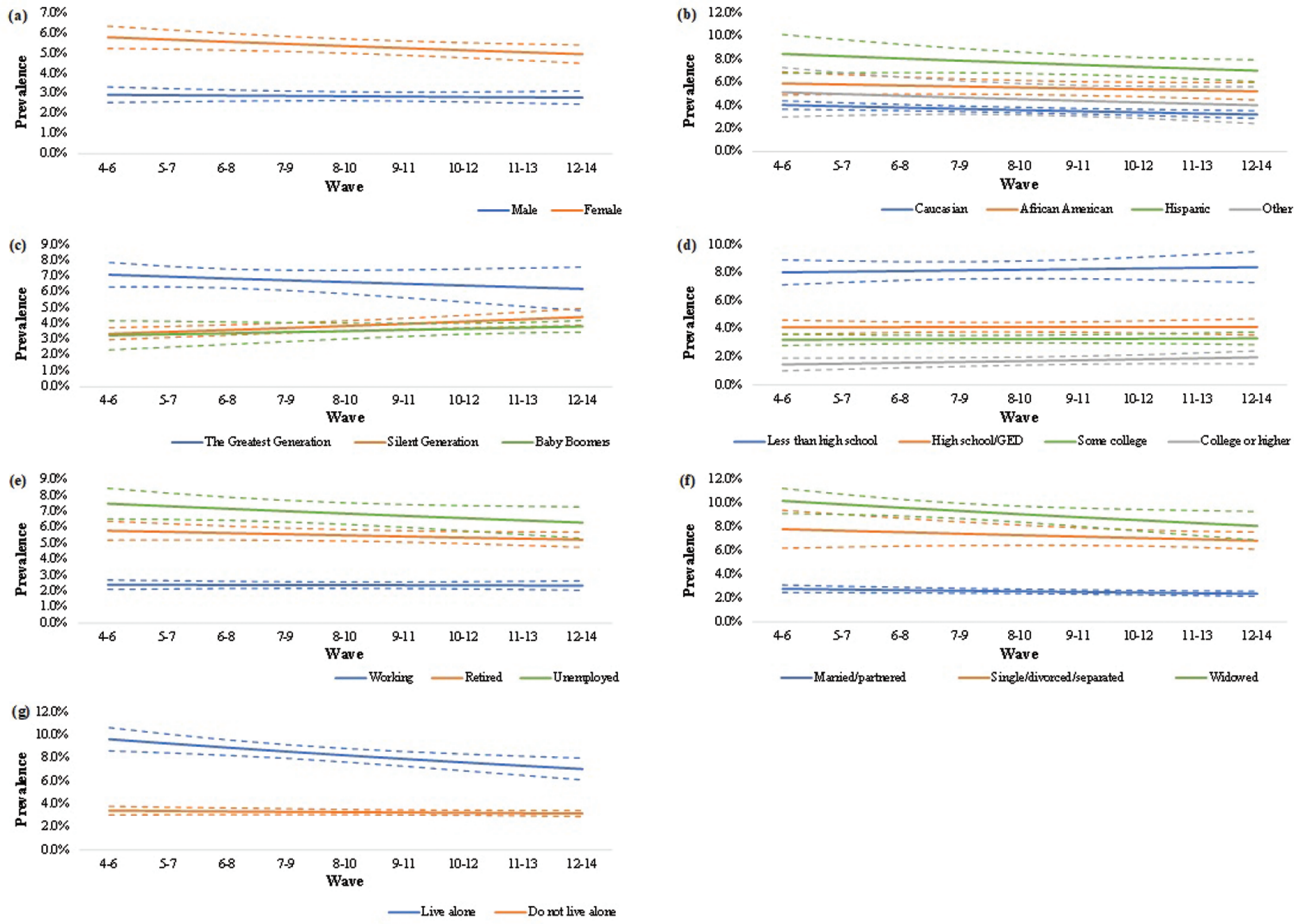
Prevalence of sustained loneliness across (A) sex, (B) race/ethnicity, (C) birth cohort, (D) education, (E) employment, (F) marital status, and (G) living alone with 95% confidence intervals.

Analysis of trends in loneliness showed decreases in both episodic (relative risk [RR]: 0.98; 95% CI: 0.98–0.99; *p* < .001) and sustained loneliness prevalence (RR: 0.98; 95% CI: 0.97–0.98) over the last 20 years. Figures 2 and 3 present the prevalence of loneliness, for episodic and sustained loneliness, respectively, by sociodemographic subgroups. Estimates for trends in each subgroup are presented in Supplementary Table S2. Overall, no wave by sociodemographic characteristic interaction was significant (*p* for interactions >.05, Supplementary Table S3), suggesting that the temporal trends within sociodemographic subgroups were not statistically different from each other.

#### Sex

Despite female participants reporting consistently higher prevalence at each wave, the episodic prevalence for loneliness decreased equally for male (RR: 0.98; 95% CI: 0.98–0.99) and female (RR: 0.98; 95% CI: 0.97–0.98) participants across the study period. This was similar for sustained loneliness among females (RR: 0.98; 95% CI: 0.96–1.00; *p* = .026); however, trends for males were not significant (RR: 0.99; 95% CI: 0.97–1.02).

#### Race/ethnicity

Caucasians consistently reported the lowest prevalence followed by “Other” and African Americans, whereas Hispanics consistently reported the highest prevalence of loneliness. Despite similar decreasing trends for both episodic and sustained loneliness across all groups, only Caucasians had significant trends for both, whereas African Americans (RR: 0.97; 95% CI: 0.96–0.98) and Hispanics (RR: 0.97; 95% CI: 0.96–0.98) only had significant trends for episodic loneliness.

#### Birth cohort

The Greatest Generation consistently reported a higher prevalence of loneliness at each wave; however, only the Silent Generation reported an increase for both episodic (RR: 1.01; 95% CI: 1.01–1.02) and sustained loneliness (RR: 1.04; 95% CI: 1.01–1.06). For both episodic and sustained loneliness, the trends for Baby Boomers (RR: 1.00; 95% CI: 0.99–1.01; RR: 1.02; 95% CI: 0.98–1.06, respectively) and the Greatest Generation (RR: 1.00; 95% CI: 0.99–1.01; RR: 0.98; 95% CI: 0.95–1.02, respectively) remained unchanged.

#### Education

Those with college or higher education consistently reported the lowest prevalence of loneliness, with an inverse association between educational level and the prevalence of loneliness. Only participants with less than high school education reported a decrease in episodic loneliness (RR: 0.99; 95% CI: 0.98–1.00; *p* = .004). All other groups reported unchanged episodic (RR ranging from 0.99 to 1.00; *p* values ranging from .127 to .597) and sustained loneliness (RR ranging from 1.00 to 1.04; *p* values ranging from .185 to .952).

#### Employment status

Working participants consistently reported the lowest prevalence of loneliness across measures over time, whereas unemployed participants consistently reported the highest. All groups showed similarly decreasing trends for episodic loneliness (RR: 0.98 for all groups with slight variations in CI), with no significant change reported for sustained loneliness (RR ranging from 0.98 to 1.00; *p* values ranging from .110 to .820).

#### Marital status

Overall, married/partnered participants had the lowest prevalence of loneliness and widowed participants had the highest. The prevalence of episodic loneliness decreased in all three groups (RR ranging from 0.97 to 0.99) and the prevalence of sustained loneliness decreased significantly in those who were married/partnered (RR: 0.98; 95% CI: 0.96–1.00; *p* = .035) and those who were widowed (RR: 0.97; 95% CI: 0.95–1.00; *p* = .037).

#### Living alone

Participants who lived alone consistently reported a higher prevalence of loneliness than those who did not. However, despite the prevalence of episodic loneliness decreasing equally for both groups (RR: 0.97; 95% CI: 0.97–0.98), only participants who lived alone reported a decrease in sustained loneliness (RR: 0.96; 95% CI: 0.94–0.99). The prevalence of sustained loneliness for participants who did not live alone remained unchanged (RR: 0.99; 95% CI: 0.97–1.01).

### Sociodemographic Predictors of Loneliness

Table 2 presents the sociodemographic predictors of both episodic and sustained loneliness based on multivariable mixed-effects Poisson models. In most cases, sociodemographic predictors had stronger associations with sustained loneliness than with episodic loneliness. Specifically, female participants were 14% more likely to report episodic loneliness than males, but 31% more likely to report sustained loneliness. African American, Hispanic, and “Other” race/ethnicity participants were 13%, 40%, and 20% more likely to report episodic loneliness, whereas Hispanic participants were 56% more likely to report sustained loneliness. Although African American and “Other” participants had an 11% and 22% higher risk for sustained loneliness, the difference was not statistically significant (*p* = .064 and *p* = .187, respectively). Compared with those with a college education, participants with some college, high school graduate/GED, and less than high school level education were 33%, 50%, and 95% more likely to experience episodic loneliness, and 60%, 93%, and 206% more likely to experience sustained loneliness. Retired and unemployed participants reported a 41% and 48% higher risk for episodic loneliness and an 88% and 96% higher risk for sustained loneliness compared to their working counterparts, respectively. Those who were single/divorced/separated and widowed reported a 62% and 71% higher risk of episodic loneliness and a 150% and 136% higher risk of sustained loneliness than their married/partnered counterparts. Finally, those who lived alone reported a 13% higher risk for both episodic and sustained loneliness than those who did not live alone; however, the latter was not statistically significant (*p* = .086). The only case where the associations trended slightly differently for episodic and sustained loneliness was regarding the birth cohort. Compared with Baby Boomers, the Silent Generation had a lower risk of reporting both episodic and sustained loneliness (13% and 23%, respectively). In contrast, the Greatest Generation had a higher risk for episodic loneliness than the Baby Boomers, but a lower risk for sustained loneliness, although the difference was not statistically significant (*p* = .180 and *p* = .133, respectively).

**Table 2.** Longitudinal Sociodemographic Predictors of Episodic and Sustained Loneliness

		Episodic loneliness		Sustained loneliness	
		RR (95% CI)	*p* Value	RR (95% CI)	*p* Value
Wave (continuous)		0.98 (0.98, 0.99)	<.001	0.98 (0.96, 1.00)	.024
Sex	Male	REF		REF	
	Female	1.14 (1.11, 1.18)	<.001	1.31 (1.19, 1.46)	<.001
Race/ethnicity	Caucasian	REF		REF	
	Not Caucasian^a^	1.22 (1.18, 1.27)	<.001	1.27 (1.14, 1.41)	<.001
	African American	1.13 (1.08, 1.17)	<.001	1.11 (0.99, 1.46)	.064
	Hispanic	1.40 (1.32, 1.48)	<.001	1.56 (1.35, 1.79)	<.001
	Other	1.20 (1.10, 1.32)	<.001	1.22 (0.91, 1.63)	.187
Birth cohort	Baby Boomers (1946–1964)	REF		REF	
	The Silent Generation (1928–1945)	0.87 (0.84, 0.91)	<.001	0.77 (0.68, 0.87)	<.001
	The Greatest Generation (1901–1927)	1.04 (0.98, 1.09)	.180	0.88 (0.75, 1.04)	.133
Education	College and above	REF		REF	
	Some college	1.33 (1.25, 1.41)	<.001	1.60 (1.32, 1.94)	<.001
	High school graduate/GED	1.50 (1.42, 1.58)	<.001	1.93 (1.61, 2.31)	<.001
	Less than high school	1.95 (1.83, 2.07)	<.001	3.06 (2.51, 3.72)	<.001
Employment status	Working	REF		REF	
	Retired	1.41 (1.37, 1.46)	<.001	1.88 (1.70, 2.08)	<.001
	Unemployed	1.48 (1.42, 1.55)	<.001	1.96 (1.75, 2.18)	<.001
Marital status	Married/partnered	REF		REF	
	Single/divorced/separated	1.62 (1.54, 1.70)	<.001	2.50 (2.14, 2.92)	<.001
	Widowed	1.71 (1.63, 1.79)	<.001	2.36 (2.04, 2.72)	<.001
Live alone	No	REF		REF	
	Yes	1.13 (1.08, 1.18)	<.001	1.13 (0.98,1.30)	.086

*Notes*: CI = confidence interval; GED = General Educational Development; REF = reference; RR = relative risk.

^a^Not Caucasian consists of African American, Hispanic, and “Other” race/ethnicity groups.

## Discussion

This is one of the first studies to examine the temporal trends of loneliness among middle-aged and older adults over time. To the best of our knowledge, this is the first study to examine the trends of both episodic and sustained loneliness. Contrary to popular belief that loneliness has become increasingly common, findings from our study provided evidence that loneliness decreased among middle-aged and older American adults between 1998 and 2018. This trend was shared by most sociodemographic subgroups with only a few exceptions. Overall, we did not find evidence to support the public perception that loneliness is increasing over time for middle-aged and older adults.

In a previous repeated cross-sectional study using the nationally representative Swedish Panel Study of Living Conditions of the Oldest Old cohort, Dahlberg et al. (2018) found that older adults reported a relatively stable prevalence of loneliness between 1992 and 2014. Another study conducted by Hawkley et al. (2019) found no evidence that loneliness increased among older American adults between 2005 and 2016. The decreasing trends in loneliness we observed may be a result of social and lifestyle changes over the past few decades, such as the increasing use of smartphones and social media which have facilitated communications. Previous evidence indicated a potential positive relationship between social media use and loneliness (Coombs, 2020); however, the association may depend on the motivation for using social media. For example, when used to strengthen current relationships and establish new connections, social media use is related to lower levels of loneliness (Nowland et al., 2017). However, if used as a tool to escape the pressures of real-life social connections, then loneliness may increase consequentially (Nowland et al., 2017). With regard to older adults, digital technologies could be a convenient and useful way for reducing social isolation (Sen et al., 2021), and some evidence does support that active use of social media could reduce loneliness (Vošner et al., 2016). Unfortunately, we did not have detailed information on technology use and communication throughout the study period to explore whether the reduced prevalence of loneliness was indeed due to smartphone and social media use.

Importantly, although this study provides a useful pre-COVID baseline for loneliness among middle-aged and older American adults, several years of living with COVID-19 may have affected the trends in loneliness at the population level. A recent study (Peng & Roth, 2021) that also used the HRS data noted that despite increased physical isolation, no change in loneliness was observed within the HRS sample between 2016 and 2020. These findings are consistent with other national studies globally which found minimal changes in loneliness before and during COVID-19 (Bu et al., 2020; Luchetti et al., 2020) where older adults seemed particularly resilient.

### Temporal Trends by Sociodemographic Subgroups

When examining temporal trends by sociodemographic characteristics, in most cases, the trends of loneliness were similar across subgroups (e.g., male vs female participants, participants of different racial/ethnic backgrounds). However, a few specific groups experienced different trends from others. For example, the Greatest Generation reported more favorable trends for sustained loneliness prevalence compared to their younger counterparts, whereas the Silent Generation reported the steepest increase in both episodic and sustained loneliness. Loneliness increasing in this specific group may be the result of the Silent Generation (aged approximately 51–68 at Wave 3 in 1996) experiencing a greater loss of social connections during the study period (e.g., children moving out of home, deaths of family and friends). However, this increase was not observed in the older cohort (the Greatest Generation, aged approximately 69–95 years at Wave 3), likely due to survival effects (i.e., those with more social resources and resilience to negative life events are more likely to survive to an older age).

Working adults consistently reported a lower prevalence of loneliness than their nonworking counterparts. This may be because those who are working have more opportunities for social connections and interactions, or perhaps the social connectivity associated with employment is what is prompting individuals to continue working into older age (Morrish & Medina-Lara, 2021). Alternatively, those who are in the workforce also tend to be healthier, and those with limiting health conditions and disabilities tend to be lonelier than their disease- and disability-free counterparts (Barlow et al., 2015; Rokach et al., 2006). With no differences in trends found across groups, we conclude that in terms of loneliness, the “advantage” of working older adults over their nonworking counterparts is likely to persist over time.

An interesting finding of our study is that despite the widowed participants starting as the most lonely marital subgroup, they tended to experience a slightly faster decrease in loneliness than their counterparts. According to the literature, people tend to experience a sharp increase in loneliness immediately after bereavement, but loneliness attenuates over time (Vedder et al., 2022). It may be that after the death of a partner, loneliness peaks and can therefore only abate going forward. Additionally, trends for those who lived alone in comparison to those who did not were largely similar to that of marital status. This is expected as the two variables are closely interlinked (but not colinear) as nearly all participants who were married/partnered did not live alone (99.2%) and they made up the majority of participants who did not live alone (83.4%).

### Predictors of Loneliness

Considering the interrelations across sociodemographic characteristics, we conducted multivariable-adjusted models to tease out the association between each sociodemographic characteristic and loneliness. Our findings suggested that during the study period (1996–2018), females, non-Caucasians, those with lower education levels, those who were retired or unemployed, those who were not married or partnered, and those who lived alone were more likely to report loneliness at both a single time point and over a sustained period. Moreover, the Silent Generation was the least likely to report episodic or sustained loneliness, while the Greatest Generation was the most likely to report episodic loneliness, and the Baby Boomers were the most likely to report sustained loneliness. These findings echoed and extended the literature by using longitudinal data analysis and examining sustained loneliness over multiple measures (Cohen-Mansfield et al., 2016; Luhmann & Hawkley, 2016). Identifying these “at-risk” subgroups helps inform targeted and tailored efforts to address the disproportionately higher prevalence of loneliness experienced by these groups.

### Strengths and Limitations

We examined trends in the episodic and sustained prevalence of loneliness across more than 20 years of longitudinal data, making this one of the largest and most comprehensive studies of its kind to date. Because HRS offers large population-representative samples after weighting, our analysis was well powered and has external validity. Examining both episodic and sustained prevalence provides new insight into population trends and predictors of both transient and chronic loneliness. To date, most studies that have examined loneliness relied on one measurement in time (Mund et al., 2019; Theeke, 2009), making it impossible to distinguish between those who sporadically experience loneliness from those who are chronically lonely. Estimating population prevalence based on a single measure of loneliness assumes that people’s experiences of loneliness remain constant over time, ignoring the sporadic and fluctuating nature of the experience of loneliness. Our findings suggest that relying on one measure could lead to overestimation of the prevalence of problematic loneliness, whereas capitalizing on multiple measures is likely to detect those who are truly “at risk.” Additionally, we observed stronger associations between sociodemographic characteristics and sustained loneliness than that with transient loneliness. This is potentially because the measure for sustained loneliness had better validity than the one for episodic loneliness.

Despite the strengths, this study is subject to several limitations. First, loneliness is measured based on a single-item question, which could lead to underreporting due to the stigma associated with loneliness, or misclassification due to different interpretations of the term “loneliness.” Bias related to this limitation may affect some subgroups disproportionately, such as male participants due to the stigma surrounding mental health (Vogel et al., 2014), or non-Caucasian participants due to language and cultural interpretation. Additionally, the use of a dichotomous response category for feeling lonely (yes/no) may have introduced additional bias by oversimplifying a complex experience. Although the HRS did include three-item UCLA Loneliness Scale items later in Wave 7, the lack of repeated data points has made the data less suitable for the purpose of the current study. Second, HRS also utilizes a mode shift in interviewing every alternating wave, which is likely to have introduced some elements of response bias, due to interviewer and social desirability bias. However, a previous study found no difference in reported loneliness prevalence between face-to-face and telephone-based interviews (Cernat et al., 2016).

### Public Health Implications

Contrary to media portrayals of increasing loneliness, we have found that since 1998, the prevalence of loneliness has decreased among middle-aged and older Americans. Some groups are at greater risk of experiencing problematic loneliness, such as females, non-Caucasians, those with lower education levels, those who were unemployed or retired, those who were not married or partnered, and those who lived alone. Considering the demographic and social trends related to the identified predictors of loneliness, such as population aging, increasing the proportion of those who are unpartnered or living alone, societies should implement surveillance systems to monitor the social health of the population and prevent exacerbation of loneliness. In our study, we also found that the prevalence of chronic loneliness is significantly lower than that of sporadic loneliness. Greater distinction should be made between these two concepts in public health surveillance and benchmarking. Chronic loneliness affects public health and needs to be addressed systematically and comprehensively as a public health issue (Ding et al., 2022).

## Supplementary Material

Supplementary data are available at *The Journals of Gerontology, Series B: Psychological Sciences and Social Sciences* online.

gbad062_suppl_Supplementary_Material
