# Supplementary material for: Have Middle-Aged and Older Americans Become Lonelier? 20-Year Trends From the Health and Retirement Study
Source: J Gerontol B Psychol Sci Soc Sci. 2023 Jun 4;78(7):1215–23. doi: 10.1093/geronb/gbad062 (PMC10292834; doi:10.1093/geronb/gbad062)
Supplement: gbad062_suppl_Supplementary_Material [file gbad062_suppl_supplementary_material.docx]

**Have middle-aged and older Americans become lonelier? 20-year trends from the Health and Retirement Study - Appendices**

**Extended Methods**

**Appendix A |** Analysis models **1**

**Appendix B |** Patterns of missing data for specific variables **3**

**Appendix C |** Imputation methods  **5**

**Supplementary Files**

**Table S1 |** The Strengthening the Reporting of Observational Studies in Epidemiology
(STROBE) reporting checklist for cohort studies **6**

**Figure S1 |** Study participant flowchart  **8**

**Table S2 |** Temporal trends of episodic and sustained loneliness within groups **9**

**Table S3 |** Relative risk (RR) for the wave by sociodemographic characteristics
 interactions with episodic and sustained loneliness as the outcomes **10**

**References 11**

**Appendix A |** Analysis models

We analyzed the data in long-format, with a unique record for each participant at each time point. Thus, there were up to 12 unique records for each participant in the study (median=6, 25^th^-75^th^ percentile: 4-10; for distribution please see the table below). To allow for temporal separation between exposure and outcome, we used lagged models, with outcomes at time 𝑡+1 regressed on exposures at 𝑡 (or, in the case of prolonged loneliness, outcomes derived from episodic loneliness at times 𝑡+1, 𝑡+2 and 𝑡+3). Thus, there were up to 11 records for each participant in the analysis of episodic loneliness, and up to 9 records for each participant for the analysis of sustained loneliness.

In order to examine not only changes in loneliness over time, but also whether those changes varied across sociodemographic characteristics, while accounting for clustering due to non-independence caused by repeated measures of the same participants, we conducted a series of Poisson regression models. Each model included main effect terms for time as a continuous variable, the relevant sociodemographic variable as a factor variable, and the time by socioeconomic variable interaction (in order to allow changes over time to vary across socioeconomic groups).

For example, the model for sex can be expressed as:

𝑙𝑜𝑔(𝑙𝑜𝑛𝑒𝑙𝑦_𝑡+1,𝑗_)~𝛽0+𝛽1×𝑡𝑖𝑚𝑒_𝑡,𝑗_+𝛽2×(*sex*_𝑡,𝑗_=𝐹𝑒𝑚𝑎𝑙𝑒)+𝛽3×𝑡𝑖𝑚𝑒_𝑡,𝑗_×(*sex*_𝑡,𝑗_=𝐹𝑒𝑚𝑎𝑙𝑒)+𝑢_𝑗_+𝑒_𝑡,𝑗_

where 𝑢𝑗 is a participant-specific random error/variance term, and 𝑒𝑡,𝑗 is an observation-specific error/variation term. Because the model estimated a single coefficient for time (and for each time by socioeconomic variable interaction), equating to the RR of a unit-increase in time (in this case corresponding to 2 years), risk ratios were then constructed using the linear combinations of the fixed-effects terms. For example, the RR for females in 2004 was constructed from:

𝑅*R*(𝑌|2004,𝑓𝑒𝑚𝑎𝑙𝑒)=exp(𝛽1×6+𝛽2×1+𝛽3×6×1),

with standard errors (and, correspondingly, confidence intervals) calculated analytically from the variance-covariance matrix (Johnson & Wichern, 2014). For sociodemographic variables with more than 2 levels (i.e. categorical variables rather than binary variables), indicator variables were used – thus, for variables with n levels, n-1 separate coefficients were estimated (i.e. a coefficient for each level of the variable except the ‘reference’ category). Thus, the model for birth cohort was:

𝑙𝑜𝑔(𝑙𝑜𝑛𝑒𝑙𝑦_𝑡+1,𝑗_)~𝛽0+𝛽1×𝑡𝑖𝑚𝑒_𝑡,𝑗_+𝛽2×(𝑐𝑜ℎ𝑜𝑟𝑡_𝑡,𝑗_=𝑆𝑖𝑙𝑒𝑛𝑡)+𝛽3×(𝑐𝑜ℎ𝑜𝑟𝑡_𝑡,𝑗_=𝐺𝐼)+𝛽4×
𝑡𝑖𝑚𝑒_𝑡,𝑗_×(𝑐𝑜ℎ𝑜𝑟𝑡_𝑡,𝑗_=𝑆𝑖𝑙𝑒𝑛𝑡)+𝛽5×𝑡𝑖𝑚𝑒_𝑡,𝑗_×(𝑐𝑜ℎ𝑜𝑟𝑡_𝑡,𝑗_=𝐺𝐼)+𝑢_𝑗_+𝑒_𝑡,𝑗_

Models were estimated using maximum likelihood estimation (ML) of the log-likelihood, with seven integral points estimated using mean-variance adaptive Gauss-Hermite quadrature (Skrondal & Rabe-Hesketh, 2004).

Analysis code used for all models is available at: <https://www.philipclare.com/code/hrs/>

Participants by the number of waves they participated in

| **Number of waves completed** | **n** | **%** |
| --- | --- | --- |
| 1 | 1,958 | 5.0 |
| 2 | 5,247 | 13.4 |
| 3 | 2,161 | 5.5 |
| 4 | 1,973 | 5.1 |
| 5 | 7,796 | 20.0 |
| 6 | 1,584 | 4.1 |
| 7 | 1,834 | 4.7 |
| 8 | 4,287 | 11.0 |
| 9 | 1,463 | 3.7 |
| 10 | 1,352 | 3.5 |
| 11 | 3,262 | 8.4 |
| 12 | 6,152 | 15.8 |
| Total | 39,069 | 100 |

**Appendix B** | 1) Patterns of missing data for specific variables

| **Variable** | **Patterns** | | | | | | | | | | | | **Number missing in variable** |
| --- | --- | --- | --- | --- | --- | --- | --- | --- | --- | --- | --- | --- | --- |
|  | **(1)** | **(2)** | **(3)** | **(4)** | **(5)** | **(6)** | **(7)** | **(8)** | **(9)** | **(10)** | **(11)** | **(12)** |  |
| Episodic loneliness | X | X | X |  | X |  |  |  | X | X |  | X | 145,144 |
| Sex |  |  |  |  |  |  |  |  |  |  |  |  | 0 |
| Race/ethnicity |  |  |  |  |  |  |  | X | X |  |  | X | 496 |
| Birth cohort |  |  |  |  |  |  |  |  |  |  |  |  | 0 |
| Education | X |  |  |  |  |  |  |  | X |  |  | X | 101,787 |
| Employment status | X | X |  | X | X |  |  |  | X |  |  | X | 131,490 |
| Marital status | X | X |  |  |  |  | X |  | X |  |  | X | 128,615 |
| Born in US^a^ |  |  |  |  |  |  |  |  | X |  |  |  | 446 |
| Religious^a^ | X |  |  |  |  | X |  |  | X |  |  | X | 102,579 |
| Lives alone^a^ | X | X |  |  |  |  |  |  | X |  |  | X | 128,436 |
| Has living children^a^ |  |  |  |  |  |  |  |  |  |  |  |  | 0 |
| Depressed^a^ | X | X | X |  | X |  |  |  | X |  | X | X | 145,135 |
| Number missing in pattern | 101,434 | 26,516 | 15,791 | 2,397 | 649 | 449 | 174 | 147 | 142 | 123 | 115 | 112 | 148,577 |

*Note.* Xs shown in the table represent that data was missing for that specific variable in that ‘pattern’ of missing data. The total number of cases with each ‘pattern’ of missing data are shown in the bottom row. The number of cases with missing data for each specific variable are shown in the right-most column.

^a^Ancillary variables in the imputation as possible indicators of retention. Not included in primary analyses.

2) Patterns of missing data due to non-participation

|  | **Wave n (%)** | | | | | | | | | | | | | |  | |
| --- | --- | --- | --- | --- | --- | --- | --- | --- | --- | --- | --- | --- | --- | --- | --- | --- |
| **Interview Status** | **2** | **3** | **4** | **5** | **6** | **7** | **8** | **9** | **10** | **11** | **12** | **13** | **14** | **Total** | |  |
| Not yet in sample | 21,042 (49.8) | 20,895 (49.5) | 15,773 (37.3) | 15,627 (37.0) | 15,467 (36.6) | 11,922 (28.2) | 11,764 (27.9) | 11,639 (27.6) | 5,028 (11.9) | 4,855 (11.5) | 4,713 (11.2) | 97 (0.2) | 0 (0.0) | 138,822 (25.3) | |  |
| Responded | 19,642 (46.5) | 17,991 (42.6) | 21,384 (50.6) | 19,578 (46.4) | 18,165 (43.0) | 20,129 (47.7) | 18,469 (43.7) | 17,217 (40.8) | 22,034 (52.2) | 20,554 (48.7) | 18,747 (44.4) | 20,912 (49.5) | 17,146 (40.6) | 251,968 (45.9) | |  |
| Non-response | 1,323 (3.1) | 2,033 (4.8) | 2,419 (5.7) | 2,931 (6.9) | 2,933 (6.9) | 3,244 (7.7) | 3,691 (8.7) | 3,774 (8.9) | 3,959 (9.4) | 4,410 (10.4) | 5,016 (11.9) | 5,985 (14.2) | 8,626 (20.4) | 50,344 (9.2) | |  |
| Died | 226 (0.5) | 1,314 (3.1) | 2,657 (6.3) | 4,097 (9.7) | 5,668 (13.4) | 6,938 (16.4) | 8,309 (19.7) | 9,603 (22.7) | 11,212 (26.5) | 12,414 (29.4) | 13,757 (32.6) | 15,239 (36.1) | 16,461 (39.0) | 107,895 (19.7) | |  |
| Total | 42,233 | 42,233 | 42,233 | 42,233 | 42,233 | 42,233 | 42,233 | 42,233 | 42,233 | 42,233 | 42,233 | 42,233 | 42,233 | 549,029 | |  |

**Appendix C |** Imputation methods

The data contained a relatively large number of missing data points, both because of attrition/intermittent completion of waves, and also because not all participants answered every question in each survey. Information about the amount and most common patterns of missing data is included in Appendix B.

Data was confirmed to be not missing completely at random via Little’s test. As such, we have assumed the data to be missing at random. Because missingness can introduce bias when there is missingness in both the outcome and exposure variables (Hughes, Heron, Sterne, & Tilling, 2019) that is not completely at random (Pedersen et al., 2017), we conducted the analyses using multiple imputation. Because some participants were entered into the study after ‘baseline’, we imputed missing study waves subsequent to the first observed wave for any participant, but not for prior waves.

We conducted the imputation using fully conditional specification (FCS; also called chained equations) in R (ver. 4.0.3) (White, Royston, & Wood, 2011). Consistent with recommendations in the literature, we first attempted to impute using the ‘just another variable’ approach (Huque, Carlin, Simpson, & Lee, 2018), treating repeated measurements as individual variables. However, this approach failed to converge. As such, we used traditional FCS, which has been shown to perform as well in longitudinal data as more complex methods (De Silva, Moreno-Betancur, De Livera, Lee, & Simpson, 2017; Huque et al., 2018). To allow for possible non-linear effects and interactions in the imputation, we imputed using random forests (Shah, Bartlett, Carpenter, Nicholas, & Hemingway, 2014).

Consistent with the principle of compatibility between the imputation and outcome models, which states that multiple imputation can introduce bias if the imputation model does not include variables or other information that is used in the outcome models (Pedersen et al., 2017), imputation models contained all variables used in the outcome models, as well as the clustering and complex survey variables. Analyses were then conducted on each imputed dataset and combined using Rubin’s rules. We used M=50 imputations to be conservative (White et al., 2011).

**Table S1 |** The Strengthening the Reporting of Observational Studies in Epidemiology (STROBE) reporting checklist for cohort studies

|  | **Item No** | **Recommendation** | **Page** |
| --- | --- | --- | --- |
| **Title and abstract** | 1 | (*a*) Indicate the study’s design with a commonly used term in the title or the abstract | Title Page |
|  |  | (*b*) Provide in the abstract an informative and balanced summary of what was done and what was found | 1 |
| **Introduction** | | |  |
| Background/rationale | 2 | Explain the scientific background and rationale for the investigation being reported | 2 |
| Objectives | 3 | State specific objectives, including any prespecified hypotheses | 3 |
| **Methods** | | |  |
| Study design | 4 | Present key elements of study design early in the paper | 4/5 |
| Setting | 5 | Describe the setting, locations, and relevant dates, including periods of recruitment, exposure, follow-up, and data collection | 4/5 |
| Participants | 6 | (*a*) Give the eligibility criteria, and the sources and methods of selection of participants. Describe methods of follow-up | 4/5 |
|  |  | (*b*) For matched studies, give matching criteria and number of exposed and unexposed | NA |
| Variables | 7 | Clearly define all outcomes, exposures, predictors, potential confounders, and effect modifiers. Give diagnostic criteria, if applicable | 5/6 |
| Data sources/ measurement | 8* | For each variable of interest, give sources of data and details of methods of assessment (measurement). Describe comparability of assessment methods if there is more than one group | 5/6 |
| Bias | 9 | Describe any efforts to address potential sources of bias | 8 |
| Study size | 10 | Explain how the study size was arrived at | 16 |
| Quantitative variables | 11 | Explain how quantitative variables were handled in the analyses. If applicable, describe which groupings were chosen and why | 6/7 |
| Statistical methods | 12 | (*a*) Describe all statistical methods, including those used to control for confounding | 6/7 |
|  |  | (*b*) Describe any methods used to examine subgroups and interactions | 6/7 |
|  |  | (*c*) Explain how missing data were addressed | 8 |
|  |  | (*d*) If applicable, explain how loss to follow-up was addressed | 8, Appendix B |
|  |  | (*e*) Describe any sensitivity analyses | NA |
| **Results** | | |  |
| Participants | 13* | (a) Report numbers of individuals at each stage of study—eg numbers potentially eligible, examined for eligibility, confirmed eligible, included in the study, completing follow-up, and analysed | Table 1 |
|  |  | (b) Give reasons for non-participation at each stage | NA |
|  |  | (c) Consider use of a flow diagram | Figure S1 |
| Descriptive data | 14 | (a) Give characteristics of study participants (eg demographic, clinical, social) and information on exposures and potential confounders | Table 1 |
|  |  | (b) Indicate number of participants with missing data for each variable of interest | Appendix B |
|  |  | (c) Summarise follow-up time (eg, average and total amount) | NA |
| Outcome data | 15 | Report numbers of outcome events or summary measures over time | Figures 1-3 |
| Main results | 16 | (*a*) Give unadjusted estimates and, if applicable, confounder-adjusted estimates and their precision (eg, 95% confidence interval). Make clear which confounders were adjusted for and why they were included | Table S2 |
|  |  | (*b*) Report category boundaries when continuous variables were categorized | NA |
|  |  | (*c*) If relevant, consider translating estimates of relative risk into absolute risk for a meaningful time period | NA |
| Other analyses | 17 | Report other analyses done—eg analyses of subgroups and interactions, and sensitivity analyses | Table 2 |
| **Discussion** | | |  |
| Key results | 18 | Summarise key results with reference to study objectives | 8-12 |
| Limitations | 19 | Discuss limitations of the study, taking into account sources of potential bias or imprecision. Discuss both direction and magnitude of any potential bias | 16/17 |
| Interpretation | 20 | Give a cautious overall interpretation of results considering objectives, limitations, multiplicity of analyses, results from similar studies, and other relevant evidence | 12-15/17 |
| Generalisability | 21 | Discuss the generalisability (external validity) of the study results | 16 |
| **Other information** | | |  |
| Funding | 22 | Give the source of funding and the role of the funders for the present study and, if applicable, for the original study on which the present article is based | 18 |

**Figure S1** | Study participant flowchart


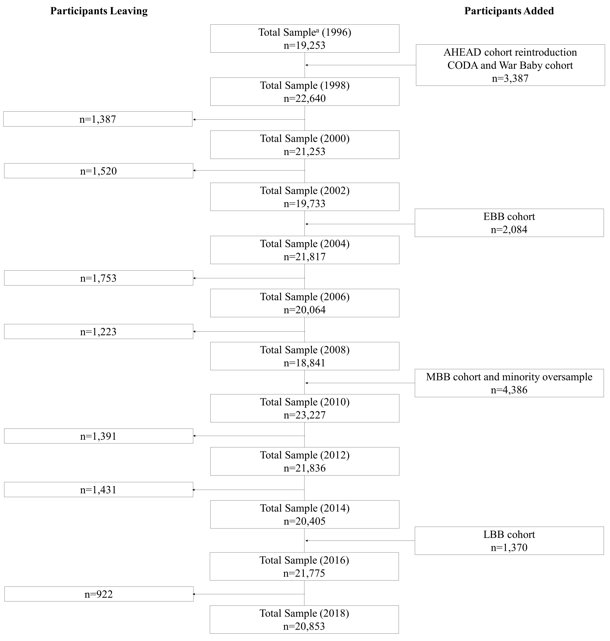


*Note.* HRS: Health and Retirement Study; AHEAD: Asset and Health Dynamics Among the Oldest Old study; CODA: Children of the Depression; EBB: Early Baby Boomer; MBB: Mid Baby Boomer; LBB: Late Baby Boomer

^a^HRS cohort only

**Table S2 |** Temporal trends of episodic and sustained loneliness within subgroups

|  |  | **Episodic Loneliness** |  | **Sustained Loneliness** |  |
| --- | --- | --- | --- | --- | --- |
|  |  | **RR (95%CI)** | ***P*-value** | **RR (95%CI)** | ***P*-value** |
| Sex | Male | 0.98 (0.98, 0.99) | *P*<0.001 | 0.99 (0.97, 1.02) | *P*=0.622 |
|  | Female | 0.98 (0.97, 0.98) | *P*<0.001 | 0.98 (0.96, 1.00^a^) | *P*=0.026 |
|  |  |  |  |  |  |
| Race/ethnicity | Caucasian | 0.97 (0.97, 0.98) | *P*<0.001 | 0.97 (0.95, 0.99) | *P*=0.003 |
|  | Not Caucasian^1^ | 0.97 (0.97, 0.98) | *P*<0.001 | 0.98 (0.96, 1.00^b^) | *P*=0.073 |
|  | African American | 0.97 (0.96, 0.98) | *P*<0.001 | 0.98 (0.96, 1.01) | *P*=0.288 |
|  | Hispanic | 0.97 (0.96, 0.98) | *P*<0.001 | 0.98 (0.95, 1.01) | *P*=0.133 |
|  | Other | 0.98 (0.95, 1.00^c^) | *P*=0.064 | 0.97 (0.90, 1.04) | *P*=0.388 |
|  |  |  |  |  |  |
| Birth cohort | Baby Boomers (1946-64) | 1.00 (0.99, 1.01) | *P*=0.909 | 1.02 (0.98, 1.06) | *P*=0.327 |
|  | The Silent Generation (1928-45) | 1.01 (1.01, 1.02) | *P*<0.001 | 1.04 (1.01, 1.06) | *P*=0.002 |
|  | The Greatest Generation (1901-27) | 1.00 (0.99, 1.01) | *P*=0.652 | 0.98 (0.95, 1.02) | *P*=0.332 |
|  |  |  |  |  |  |
| Education | College and above | 1.00 (0.98, 1.01) | *P*=0.597 | 1.04 (0.98, 1.10) | *P*=0.185 |
|  | Some college | 1.00 (0.99, 1.01) | *P*=0.490 | 1.00 (0.98, 1.03) | *P*=0.756 |
|  | High school graduate/GED | 0.99 (0.99, 1.00^d^) | *P*=0.127 | 1.00 (0.97, 1.03) | *P*=0.952 |
|  | Less than high school | 0.99 (0.98, 1.00^e^) | *P*=0.004 | 1.01 (0.98, 1.03) | *P*=0.649 |
|  |  |  |  |  |  |
| Employment | Working | 0.98 (0.98, 0.99) | *P*=0.001 | 1.00 (0.97, 1.02) | *P*=0.820 |
| status | Retired | 0.98 (0.98, 0.99) | *P*<0.001 | 0.99 (0.97, 1.01) | *P*=0.165 |
|  | Unemployed | 0.98 (0.97, 0.99) | *P*<0.001 | 0.98 (0.95, 1.01) | *P*=0.110 |
|  |  |  |  |  |  |
| Marital status | Married/partnered | 0.98 (0.97, 0.99) | *P*<0.001 | 0.98 (0.96, 1.00^f^) | *P*=0.035 |
|  | Single/divorced/separated | 0.99 (0.98, 1.00^g^) | *P*=0.019 | 0.98 (0.96, 1.01) | *P*=0.265 |
|  | Widowed | 0.97 (0.96, 0.98) | *P*<0.001 | 0.97 (0.95, 1.00^h^) | *P*=0.037 |
|  |  |  |  |  |  |
| Live alone | No | 0.97 (0.97, 0.98) | *P*<0.001 | 0.99 (0.97, 1.01) | *P*=0.257 |
|  | Yes | 0.97 (0.97, 0.98) | *P*<0.001 | 0.96 (0.94, 0.99) | *P*=0.003 |
| *Note.* Based on Poisson regression models for both loneliness outcomes, with a model for each sociodemographic variable. We used lagged models with data from one wave predicting loneliness over the subsequent wave(s). Relative risk (RR) can be interpreted as incremental change in risk of being lonely as compared with the previous wave.  Borderline confidence interval values: ^a^0.998; ^b^1.002; ^c^1.001; ^d^1.002; ^e^0.996; ^f^0.998; ^g^0.998; ^h^0.998 ^1^Not Caucasian consists of African American, Hispanic, and “Other” race/ethnicity groups. | | | | | |

**Table S3 |** Relative risk (RR) for the wave by sociodemographic characteristics interactions with episodic and sustained loneliness as the outcomes

|  |  | **Episodic Loneliness** |  | **Sustained Loneliness** |  |
| --- | --- | --- | --- | --- | --- |
|  |  | **RR (95%CI)** | ***P*-value** | **RR (95%CI)** | ***P*-value** |
| Sex | Male | REF |  | REF |  |
|  | Female | 0.99 (0.96, 1.02) | *P*=0.389 | 0.99 (0.98, 1.00) | *P*=0.129 |
|  |  |  |  |  |  |
| Race/ethnicity | Caucasian | REF |  | REF |  |
|  | Not Caucasian^a^ | 1.01 (0.98, 1.04) | *P*=0.419 | 1.00 (0.99, 1.01) | *P*=0.514 |
|  | African American | 1.01 (0.98, 1.05) | *P*=0.419 | 1.00 (0.98, 1.01) | *P*=0.422 |
|  | Hispanic | 1.01 (0.97, 1.04) | *P*=0.772 | 1.00 (0.99, 1.01) | *P*=0.510 |
|  | Other | 1.00 (0.93, 1.08) | *P*=0.955 | 1.00 (0.98, 1.03) | *P*=0.852 |
|  |  |  |  |  |  |
| Birth cohort | Baby Boomers (1946-64) | REF |  | REF |  |
|  | The Silent Generation (1928-45) | 1.01 (0.97, 1.06) | *P*=0.524 | 1.01 (0.97, 1.06) | *P*=0.524 |
|  | The Greatest Generation (1901-27) | 0.96 (0.91, 1.02) | *P*=0.179 | 0.96 (0.91, 1.02) | *P*=0.179 |
|  |  |  |  |  |  |
| Education | College and above | REF |  | REF |  |
|  | Some college | 0.97 (1.10, 1.03) | *P*=0.289 | 0.97 (1.10, 1.03) | *P*=0.289 |
|  | High school graduate/GED | 0.96 (0.91, 1.03) | *P*=0.259 | 0.96 (0.91, 1.03) | *P*=0.259 |
|  | Less than high school | 0.97 (0.91, 1.03) | *P*=0.312 | 0.97 (0.91, 1.03) | *P*=0.312 |
|  |  |  |  |  |  |
| Employment | Working | REF |  | REF |  |
| status | Retired | 0.99 (0.96, 1.02) | *P*=0.469 | 0.99 (0.96, 1.02) | *P*=0.469 |
|  | Unemployed | 0.98 (0.95, 1.02) | *P*=0.268 | 0.98 (0.95, 1.02) | *P*=0.268 |
|  |  |  |  |  |  |
| Marital status | Married/partnered | REF |  | REF |  |
|  | Single/divorced/separated | 1.00 (0.97, 1.04) | *P*=0.839 | 1.00 (0.97, 1.04) | *P*=0.839 |
|  | Widowed | 0.99 (0.96, 1.02) | *P*=0.564 | 0.99 (0.96, 1.02) | *P*=0.564 |
|  |  |  |  |  |  |
| Live alone | No | REF |  | REF |  |
|  | Yes | 0.97 (0.94, 1.00) | *P*=0.051 | 0.97 (0.94, 1.00) | *P*=0.051 |
| *Note.* Based on Poisson regression models for both loneliness outcomes, with a model for each sociodemographic variable. We used lagged models with data from one wave predicting loneliness over the subsequent wave(s). Relative risk (RR) can be interpreted as the relative difference in the trend of each group compared with the trend of the comparison group.  ^a^Not Caucasian consists of African American, Hispanic, and “Other” race/ethnicity groups. | | | | | |

**References**

De Silva, A. P., Moreno-Betancur, M., De Livera, A. M., Lee, K. J., & Simpson, J. A. (2017). A comparison of multiple imputation methods for handling missing values in longitudinal data in the presence of a time-varying covariate with a non-linear association with time: a simulation study. *BMC Med Res Methodol, 17*(1), 114. doi:10.1186/s12874-017-0372-y

Hughes, R. A., Heron, J., Sterne, J. A. C., & Tilling, K. (2019). Accounting for missing data in statistical analyses: multiple imputation is not always the answer. *International Journal of Epidemiology, 48*(4), 1294-1304. doi:10.1093/ije/dyz032

Huque, M. H., Carlin, J. B., Simpson, J. A., & Lee, K. J. (2018). A comparison of multiple imputation methods for missing data in longitudinal studies. *BMC Medical Research Methodology, 18*(1), 168. doi:10.1186/s12874-018-0615-6

Johnson, R. A., & Wichern, D. W. (2014). *Applied multivariate statistical analysis* (Vol. 6): Pearson London, UK:.

Pedersen, A. B., Mikkelsen, E. M., Cronin-Fenton, D., Kristensen, N. R., Pham, T. M., Pedersen, L., & Petersen, I. (2017). Missing data and multiple imputation in clinical epidemiological research. *Clin Epidemiol, 9*, 157-166. doi:10.2147/clep.S129785

Shah, A. D., Bartlett, J. W., Carpenter, J., Nicholas, O., & Hemingway, H. (2014). Comparison of random forest and parametric imputation models for imputing missing data using MICE: a CALIBER study. *Am J Epidemiol, 179*(6), 764-774. doi:10.1093/aje/kwt312

Skrondal, A., & Rabe-Hesketh, S. (2004). *Generalized latent variable modeling: Multilevel, longitudinal, and structural equation models*: Chapman and Hall/CRC.

White, I. R., Royston, P., & Wood, A. M. (2011). Multiple imputation using chained equations: Issues and guidance for practice. *Statistics in Medicine, 30*(4), 377-399. doi:<https://doi.org/10.1002/sim.4067>
